# Wearables for tracking mental state in the classroom: ethical considerations from the literature and high school students

**DOI:** 10.3389/fnrgo.2025.1536781

**Published:** 2025-05-13

**Authors:** Anke Snoek, Anne-Marie Brouwer, Ivo V. Stuldreher, Pim Haselager, Dorothee Horstkötter

**Affiliations:** ^1^Psychiatry, Amsterdam University Medical Center, Amsterdam, Netherlands; ^2^Human Performance, Netherlands Organisation for Applied Scientific Research (TNO), Soesterberg, Netherlands; ^3^Donders Institute for Brain, Cognition and Behaviour, Radboud University, Nijmegen, Netherlands; ^4^Health, Ethics and Society, Maastricht University, Maastricht, Netherlands

**Keywords:** wearables, physiology, education, ethics, Foucault, empirical bioethics, mental state tracking

## Abstract

**Introduction:**

Educational practice increasingly makes use of technology to improve teaching and learning. New wearable technology is being developed that measures mental states like attention and stress, through neurophysiological signals like electroencephalography (EEG), electrodermal activity (EDA) and heart rate. However, little is known about the ethical aspects of this technology.

**Methodology:**

We provide an overview of current ethical considerations on such wearable technologies in classroom settings and analyze these critically. We distinguished three ethical angles to analyze new technologies: epistemic, principle-based, and Foucauldian. We focus on a Foucauldian analysis, outlining how such technologies affect power relationships and self-understanding, but also which responses people develop to evade power. In addition, a focus group of high school students was set up to identify young people's views on such wearable technology and to initiate a reflection on the theory-based ethical considerations.

**Results:**

Our study shows that although wearables may provide information on learning and attention, and even though possible users are enthusiastic about the potential, there are several risks of applying such technologies in educational settings. These risks concern governance and surveillance, normalization and exclusion, placing technology before pedagogy, stimulating neoliberal values and quantified self-understanding, and possible negative impact on identity for those who think they are outside of the norm. High school students highlighted that people are not only subjected to new technologies, but also subject these technologies to their own goals.

**Discussion:**

We end with a discussion on the perils of implementing new technologies, and provide an alternative to prohibition in the form of co-creating and educating. Any potential future implementation of mental state tracking technology is to be accompanied by normative discussions on legitimate aims, on rights, interests and needs of both pupils, teachers, and educational institutions, taking broader debates on what should count as a good pedagogical climate into account.

## 1 Introduction

Technologies are increasingly used in educational practices. For example, many schools in the Netherlands and other Western countries, use laptops with interactive learning programs or smartboards rather than traditional books or blackboards. In addition, a variety of wearables are making their entrance into the educational setting (Motti, 2019).

In the broadest sense, the word “wearable” is used for all technologies that can be worn on the body, e.g. integrated in bracelets, glasses or clothing. For example, Nakasugi and Yamauchi (2002) developed a head mounted see-through display that allows history students to acquire historical images of today's sceneries. Google glass is a so-called smart-glass that enables students to handsfree record lectures by voice control (Coffman and Klinger, 2015). Google glass as well as the virtual reality headset of “oculus rift” are used for simulation based training, for example for medical students (Wu et al., 2014).

Often, the word “wearable” refers to technologies that are not only wearable, but that also automatically and non-intrusively collect data about the wearer, for feedback purposes, so-called tracking devices (Attallah and Ilagure, 2018; Bower and Sturman, 2015; Engen et al., 2017). This holds for smartwatches that count user's steps, heartrate, other physiological parameters but also location and social contacts. In the context of educational settings, for example, Ensmann (2020) found that such tracking devices, like *fitbits*, could improve physical activity in high schools, which in turn had a positive effect on students' cognitive functioning in the classroom.

The focus of our paper are a subtype of these (future) wearables that collect physiological data with the aim to estimate and track mental states in the classroom. These wearables measure neurophysiological signals like electroencephalography (EEG), electrodermal activity (EDA), and heart rate, and aim to translate these data to mental states and processes that are considered relevant in educational setting, like stress, or attention. The idea is that the neurophysiology underlies the mental states. While gathering physiological data is comparatively easy, to interpret such data with respect to mental states is hard, especially when individuals are in uncontrolled, non-experimental, environments. However, several studies, focusing on educational or education-like settings, suggest that inferring mental states from neurophysiological data is possible (Koide-Majima et al., 2024; Dikker et al., 2017; Poulsen et al., 2017; Rybář and Daly, 2022). For instance, Stuldreher et al. (2020) showed that attentional engagement is associated with the degree to which physiological signals of individuals fluctuate over time in the same way (interpersonal physiological synchrony). Dikker et al. (2017) have already used wearable EEG headsets in a classroom setting to predict classroom engagement. These type of wearables are currently undergoing rapid development.

Such upcoming mental state tracking devices in educational settings go together with a series of hopes, expectations and educational or pedagogical aims (see Table 1 for an overview of affordances). Data on differences in students' cognitive focus and engagement, at individual or group level, could provide insight into different learning styles or different responses to various teaching styles (Apicella et al., 2022). The hope is that such insights could be invoked to adapt teaching and teaching styles such that both students and teachers achieve are more engaged, satisfied and yield better overall outcomes (see also for example Geršak et al., 2022; Attallah and Ilagure, 2018; Bower and Sturman, 2015; Borthwick et al., 2015; Demir and Demir, 2017; Jovanovic and Kay, 2021). If used in classroom settings, such real-time physiological data on student's level of engagement couldmight even support diagnosis of attentional disorders, such as ADHD, and enable personalized intervention (Apicella et al., 2022). The hope is further that during (e.g., pandemic enforced) online and other distant forms of learning, attention tracking in students could provide students and teachers alike with more real-time insights on current attention and any fluctuations, potentially allow parties to invest in personally adapted extra support for more effective learning (Vandenbroucke et al., 2021).

**Table 1:** Overview of affordances of (mental state) tracking wearables in education.

**Affordance**	**Reference**
**Valuable data for educators/research**	
– Educators can collect valuable data on their students' cognitive focus and engagement – Improved feedback for students	Attallah and Ilagure, 2018; Bower and Sturman, 2015; Demir and Demir, 2017
– *In situ* contextual information and *in situ* guidance – Interact with the environment more naturally	Bower and Sturman, 2015; Sandall, 2016; Demir and Demir, 2017; Janssen et al., 2021
– Access information easily	Sandall, 2016
**Personalized learning**	
– Personalized learning: Data collected enables the building of student profiles—for personalized assessment and instruction. – Enhancing differentiation of instruction	Borthwick et al., 2015; Jovanovic and Kay, 2021; Geršak et al., 2022
– Extra support for students with disabilities	Attallah and Ilagure, 2018; Borthwick et al., 2015; Sandall, 2016; Demir and Demir, 2017
**Engagement**	
– Increasing student engagement and relevance	Attallah and Ilagure, 2018; Bower and Sturman, 2015; Borthwick et al., 2015; Engen et al., 2017; Sandall, 2016; Jovanovic and Kay, 2021
– Gamification: achieve a learning outcome via gamification	Demir and Demir, 2017; Bower and Sturman, 2015; Ensmann, 2020; Jovanovic and Kay, 2021
**Improve performance (students)**	
– Cross-disciplinary possibilities	Engen et al., 2018
**Empowerment of students**	
– Providing students with voice, ownership of learning and reflection	Jovanovic and Kay, 2021
– Building social presence	Jovanovic and Kay, 2021

The development and potential use of such mental tracking technologies in classrooms, either on-site or remotely, however, does not only provide hope for more effective learning and overall support to teachers and students, but also raises a series of questions regarding their social and ethical implications and the contexts in which their implementation might be justified or desirable. The aim of the current paper is to explore these ethical implications of wearables that track mental states in ways that are, potentially, employable in classroom settings. To this end, we conduct a critical analysis of current ethical literature on the topic which was further informed by insights gained during a focus group meeting with high school students who were invited to reflect on the implications of such a technology.

## 2 Three angles to conduct an ethical analysis: epistemic, principles, and Foucauldian

Literature was searched using a combination of the following keywords: ethical OR ethics OR moral, AND wearables, AND classroom OR education. We searched in Pubmed, Google scholar, Scopus, and Web of Science. We searched between 2000–2024. But as wearable technology is relatively new, most papers are from 2015–2024. We started with a scoping review. As we found very little literature on *attention tracking* wearables, we extended our search to wearables that track physiological data. We then narrowed our search down to papers that included a substantial ethical analysis of wearables in the classroom. We used a snowballing technique on the bibliographies of these papers to identify other relevant papers on ethical viewpoints. Our literature analysis is extended, using multiple strategies to identify ethical papers.

**Figure 1:**
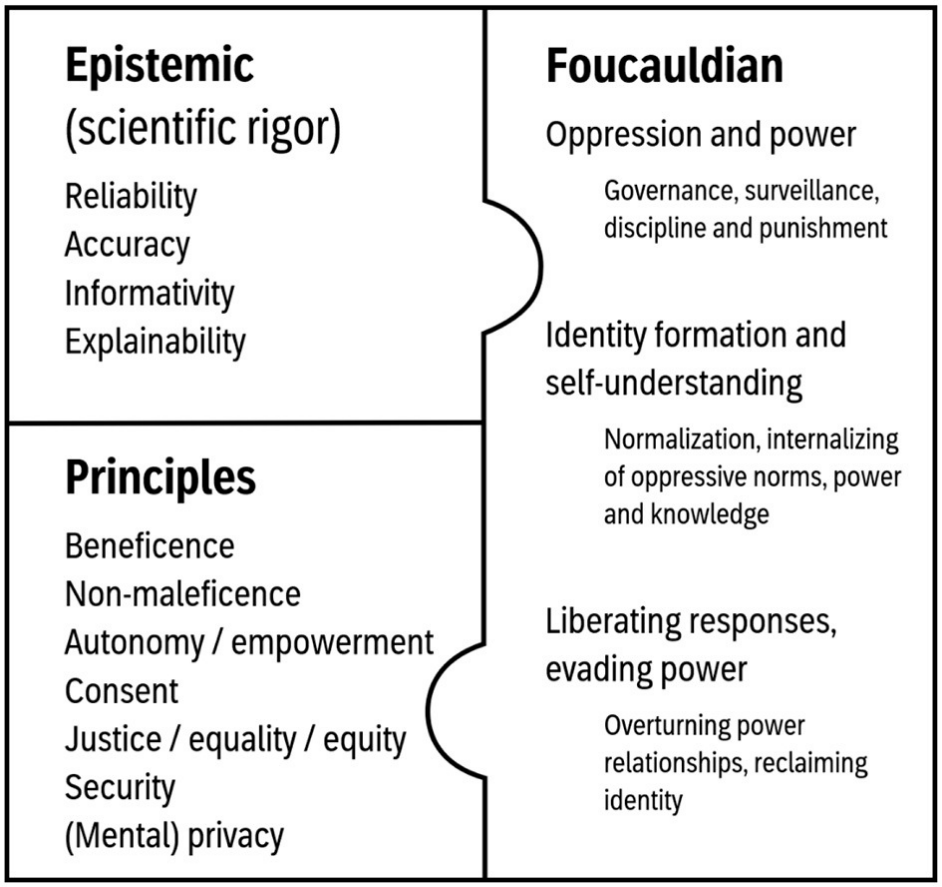
Three types of ethical analysis.

We identified ethical issues that could be grouped along three different angles of ethical analysis (Figure 1): (1) An epistemic approach that discusses the implications of the validity and reliability of the technology; what exactly is and is not measured, and how to interpret the collected data, (2) a principle-based approach that identifies which ethical principles are violated or supported, how and why, and (3) a Foucauldian approach that enables an analysis on how wearable technology for tracking mental states in the classroom influence power structures, intra- and inter-individual relationships and the identity of students and teachers.

### 2.1 Epistemic angle

The epistemic ethical approach (e.g., Maxwell and Racine, 2011) focuses on the implications of the scientific rigor, such as reliability (consistency) and accuracy (precision), of any emerging technology and argues that epistemic and ethical issues are closely intertwined. Most importantly, this approach aims to assess whether end-users have a meaningful understanding of the technology they are using, including an adequate understanding of the technology's limitations regarding, e.g., informativity and explainability (Mecacci and Haselager, 2017). In our case, ethical questions involve how accurate and reliable these wearables indeed do measure mental states. Commercial wearables are often overselling their merits (Wexler, 2019; Wexler and Thibault, 2018) and measure less than promised. However, a technology could also be underselling, and measure more than the pre-defined goals, and hence measuring more than people consented for (Borthwick et al., 2015). In our case wearables could measure not merely physiological parameters that indicate attention, but thereby, and potentially by measuring the same parameters, also provide data that allow for analysis beyond mere cognitive attention.

Another issue regards whether users understand the limits of the validity of the technology sufficiently well. For instance, if teachers unreservedly trust the physiological sensor indicating that a student regularly has attention deficits, they might be biased toward that student and wrongly judge their performance. Explainability is important in this regard (Khosravi et al., 2022). The requirement of explainability refers to how the inferential processes performed by a technology can be made understandable for human users, so that they can grasp and evaluate how certain decisions or results were reached or could be justified. A technology that is explainable to users should enhance their ability to interpret the data correctly and collaborate with a technology in a fruitful manner. Crucial in this regard, is the communication between scientists and end-users regarding the limitations and uncertainties inherent in technology usage (Brouwer, 2021).

The ideal of informativity also includes aspects of the practical relevance of the collected information. Do teachers really need the information provided by the wearables to gain insight into the attention paid by their students, or are they already able to estimate their students' concentration and attention by other means? In case a device does not provide additional insights on what is available already, this impacts, if not undermines, the ethical justifiability of implementing it. Although epistemic issues get quite some attention in technical literature on wearables, they seem largely absent in most ethical discussions. This is problematic, because questions on validity, reliability, explainability and informativity have clear ethical implications. The more valid and informative a technology is, the more justifiable is its implementation—provided safety—on top of any currently available means. At the same time, a device that appears valid and informative, but is non-transparent and hence does not live up to requirements of explainability, is more difficult to implement ethically, as it requires that users trust an unknown and untransparent source of information that they also cannot negotiate with, ask for reasons or blame in case of resulting problems. This would undo a user's agency, which, however, is of great normative significance.

### 2.2 Ethical principles angle

Secondly, the most well-known way of conducting ethical analysis is principle-based. Beauchamp and Childress, for example, have identified four fundamental ethical principles that any medical action must live up to: beneficence (doing good), non-maleficence (to do no harm), autonomy, and justice (Beauchamp and Childress, 2002). Other important principles are, for example, security, privacy, and consent. The ethical question is whether a new technology is able to support important ethical principles or also comes with risks of violating them.

For example, in the context of attention monitoring wearables in education, the expected high costs of wearables might increase inequality regarding the access of good learning tools, and compromise justice (Attallah and Ilagure, 2018; Borthwick et al., 2015; Motti, 2019; Peace et al., 2024; Sandall, 2016). With regard to autonomy, a two-sided picture emerges. One the one hand wearables might empower students, in so far as they provide them with better insight on their own mental state and allow them to gain ownership of their learning (Jovanovic and Kay, 2021). On the other hand, in case wearables are used collectively in classroom settings, their usage might be experienced as being pressured upon them, leaving little or no space for freedom of usage or rejection. The protection of privacy becomes an urgent topic in case data gets shared or uploaded to the cloud, while it remains unclear who might have access to the data collected by wearables (Attallah and Ilagure, 2018; Almusawi et al., 2021; Bower and Sturman, 2015; Borthwick et al., 2015; Motti, 2019; Demir and Demir, 2017; Engen et al., 2017; Ba and Hu, 2023). The violation of privacy is especially concerning since minors are involved, who often do not—yet—have a say on their own data, and who are often unaware of the risks involved with their data being collected and potentially accessed by third parties. In addition students might have a legitimate interest to protect data about their own mental state from their teacher. A device that continuously gathers and shares such data, could undermine students' right to protect their privacy to hitherto unknown extents. In case that mental-state tracking devices might enable “mind reading” (Haynes, 2011), it might be required to not only protect one's data, but add a new category to the ethical ideal of privacy and actual privacy regulations, concerning the protection of our *mental* privacy, or the right to keep our mental states to ourselves (Mecacci and Haselager, 2017).

### 2.3 Foucauldian ethics angle

The third angle on ethical analysis is Foucauldian. Foucault (1977) outlined that new technologies are not only devices we turn on and off, they also have to potential to change our power relationships and self-definition or identity (Foucault, 1977, 1988). As Heidegger, one of Foucault's teachers has outlined, “technology had less to do with tools, instruments, and machinery than with a particular (utilitarian) mindset or ‘attitude' that pervaded every aspect of human life” (cited in Hernández-Ramírez, 2017). Bower and Sturman (2015) summarize it as follows: “technology [functions] not only as an amplifier of cognition but also as a reorganizer of mental functioning that results in cultural redefinition.” (Bower and Sturman, 2015, p. 352). Technologies can change us on a fundamental level and influence our identity.

Three concepts are important with regard to a Foucauldian analysis.

1) **Oppression** and power: technologies can be oppressive, they and contribute to governance, surveillance, discipline and punishment.
2) **Identity**: technologies can influence how we define ourselves, they can contribute to a process of normalization and internalizing of oppressive norms.
3) **Liberating responses**, evading power: people are not only oppressed by technologies, or internalize oppressive norms, they also develop strategies and liberating responses to overturn power relationships. Power is not a one way, top down relationship, but fluid. Power happens in relationships rather than it being a property of one group that governs another group.^1^

An analysis through a Foucauldian ethics angle focusses on power-relationships and is strongly contextual. Hence, it can also provide a more layered understanding of the epistemic and ethical principles mentioned earlier. Which power dynamics are there in epistemic issues, for example, who gets to define scientific parameters? Or; how can autonomy be defined when people internalize certain (neoliberal) norms of productivity and normality? We must add that while we use some of his core concepts to analyze ethical issues of technology, this analysis is based on *our* interpretation of his work. Hence, in some ways it departs from a close reading of Foucault, who compared, for example, schools with prisons.

Before we give an overview of the literature from a Foucauldian ethics angle, we will present the data from the focus group, as this neatly illustrates what the added value is of analyzing ethical problems though the angle of power relationships, identity and liberating responses. In the Appendix, we added an overview of the ethical issues identified in the literature, grouped along the three angles: epistemic, principle-based and Foucauldian (see Appendix Table 2).

## 3 Focus group on affordances and ethical implications of wearables to track attention in education

The focus group was conducted in one class of 31 students and a teacher of a Gymnasium high school in the Netherlands. The students were between 16–17 years old, and representative with regard to gender. They were from a relatively high social economic class. The focus group was conducted by two researchers developing wearable devices (AB and IS), and one ethicist (AS). The focus group was audio recorded and transcribed ad verbatim. The data was analyzed through a grounded theory methodology in the software program Nvivo (Wertz et al., 2011).

The researchers presented future wearable attention tracking technology to the students, describing it as wrist bands that measure heart rate and skin conductance of students and therewith estimating the general attention level of the students during the class, or drop of attention in single students (see Appendix: presentation of wearables). We asked about their first impression. The first impression of the class was quite positive: 11 students thought this should be used in the classroom, 19 were neutral, and one was against using the technology. We then asked about the risks and benefits they saw regarding the technology. The Foucauldian perspective proved helpful in interpreting the data from the focus group in terms of how new technologies change power relationships and identity.

### 3.1 Students on the influence of wearable on self-image, pressure, and stress

Students outlined that wearables could negatively influence their self-image.

I think it can make people insecure. That you will compare yourself to others, and notice that you are less concentrated than others, it could have a negative effect on your self-image.

When students notice that they are less concentrated than others, it could be a source of stress, and put pressure on individuals:

I think it puts a tremendous amount of stress on the individual to be concentrated all the time. Maybe due to this pressure, you will feel stressed. On group level it could work, but on an individual level, it is a huge amount of pressure.

Students wondered whether the wearables could also measure stress levels of students. Then, in a more private setting, teachers could ask these students what they can do to make their lessons less stressful to them. Paradoxically, then the wearables that induce stress could then be used to reduce the stress levels. They emphasized that it was crucial that the teachers used the data in a benevolent way for their students.

Also, students questioned whether wearables would improve the way teachers respond to certain attention-deficit conditions.

Teachers may not always understand ADD well enough to understand what causes lack of attention, and then they do not respond adequately.

The students stated that they wanted to be understood as a person, not through the lens of a possible disorder.

### 3.2 Students fearing oppression and governance

Students worried about oppression and governance, especially the loss of their mental privacy, e.g., when malevolent teachers would misuse the data. Students expressed fears of being chipped, or having their mind read. They argued that this technology might threaten their right to not pay attention and have fun at school.

If a student does not want to pay attention, it is their right. Students are old and wise enough to determine whether they want to pay attention or not.

It's great to have fun at school.

An issue students raised that is not mentioned in the literature, is that they fear that teachers might misuse the data to pick on them in the classroom.

Some teachers may not use this to improve their lessons, but to single out individuals, and ridicule them.

A fear related to this, is that teachers might use the data to blame the students for lack of concentration, rather than reflecting on their own teaching style and how they failed as educators. In the literature, teachers are presumed to be benevolent, the students pointed out the possibility that teachers can also be malevolent.

The teacher mentioned that he thought wearables could play an important role in self-regulation, and not so much in top down regulation by the teacher. That way, the mental privacy of students would be ensured, while they could still use the benefits. The self-regulation would entail that students get a signal when they are distracted, at that moment, so that they can decide whether they want to focus or not. Overall, Foucault's ideas regarding the relation between technology and governmentality and oppression provide a way to understand the students' concerns about how wearables might undermine their self-determination during classes.

### 3.3 Students overturning power-relationships

One surprising finding of our focus group, was that students overturned the view that wearables give insight in their functioning, and stated that the data mostly gave insight in the functioning of their teachers. Hence, they saw the wearables as a way to govern and surveil inexperienced teachers. Students saw the wearables mainly as a tool to help inexperienced teachers to improve their teaching style. Students would benefit from this, because the lessons would be less boring. Rather than perceiving their own mental state as something that could be improved, students focused on using the technology to improve their teachers' capabilities. They differentiated between experienced and inexperienced teachers. For experienced teachers, they judged pedagogical qualities to be sufficient, and not in need of technological support, but for inexperienced teachers, technology could support the development of their pedagogical skills.

A good teacher knows when to intervene, but that is not the case for every teacher. I know quite a few teachers who could use this.

This is useful for new teachers, so they can see when the class zones out.

Some felt compassion for teachers who might get negative feedback: “For a teacher it can be demotivating to see that people do not pay attention in his class.” Others thought it was the teacher's own fault.

Some students also raised concerns on whether the teachers could be governed through the wearables. They remarked that there seemed to be an expectation that lessons should always be engaging and stimulating, whereas they thought that it cannot be avoided that some lessons are boring or distressing, and they should or could not always be made entertaining. Focusing too strongly on quantitative data on attention might lead to a failure to grasp this nuance.

Examining the focus group through the lens of Foucault's idea of the fluidity of power illustrates how students can not only be oppressed by technologies, but also can overturn the power technologies to improve their own position.

### 3.4 Take home message from the focus group

In general, the students were curious and positive about the technology. The students' comments nicely illustrated under which conditions, and for which groups the wearables could provide useful information. For inexperienced teachers, the technology can support them to improve their pedagogical qualities. Experienced teachers would not need this technology. When malevolent teachers use wearables, it could be very oppressive for students and it might be used to humiliate them. Students that easily feel stress might feel extra pressured due to the technology, but the technology could also be used to detect when students feel stressed, so the teacher can support them. Mostly, the focus group shows the sensible and unexpected ideas end users have about the added value and ethical risks of technologies within a specific context. The data shows that students did not feel too vulnerable with regard to new technologies, but rather have ideas on how the technology can be used to their benefit. For them, wearables are especially useful not to give insights in their own functioning, but in the functioning of their teachers.

## 4 Foucauldian ethics angle in the literature

As the data from the focus group showed the merit of an ethical analysis through the lens of power relationships and identity, we specifically analyzed the ethical data through this lens. We found in the literature mostly a focus on oppression and power, and the influence on identity, and less on the liberating responses.

### 4.1 Oppression and power: governance, surveillance, discipline and punishment through technology

Wearable technologies can be oppressive and play a role in surveillance, disciplining and punishing students. In 2019 the Wall Street Journal published a reportage about the use of wearables in Chinese classrooms such as the Jinhua Xiaoshun county primary school (Tai, 2019). At that school a variety of technologies are used in the classroom: headbands that measures brain activity, artificial-intelligence cameras, robots that analyze students' health and concentration, location trackers in the uniforms, all accompanied by a state-wide network of facial recognition by public camera's. The information gathered in the classroom on each individual student is send to teachers and parents, who then exactly know where a student is in the building, how they behave and when a student is not paying attention. The Wall Street Journal evaluates this setting as follows: “While many parents and teachers see them as tools to improve grades, they have become some children's worst nightmare.” Teachers interviewed for the reportage appear enthusiastic about the positive effect of the technology: students are more focused, they pay better attention, and have higher scores than before the installation of the devices. Some students are also enthusiastic and talk about their higher grades. But other students feel pressured and controlled. The technology puts an increased pressure on students to have high scores. A student who keeps dozing off in class reports that his parents punish him for this. Hence, the already asymmetric power relationship between teachers and parents on the one hand, and children at school on the other, gets further intensified through a technology that monitors children's mental states, reports these to teachers and parents, facilitating data-based punishment of schoolchildren.^2^

### 4.2 Identity: influence of technology on identity, self-definition, normalization and internalizing external (neoliberal) norms

#### 4.2.1 Normalization vs. diversity

Wearables can influence how people define themselves, whether they see themselves as being normal or not. When the data collected through wearables shows that someone is outside the norm, this does not necessarily mean that they malfunction, the data could also indicate a different, but effective, learning style. However, a hasty interpretation of the data, either by the teacher, student or parent, could lead to a conclusion about malfunctioning, and can influence how the student perceives himself. Teachers might not be trained enough to provide careful, non-stigmatizing feedback. Wearables can give important information on learning, but there is a risk that wearables create new, unjust categories of what normal and abnormal learning is. Students, teachers and parents might rely too heavily on raw quantified data to define learning styles, rather than look at learning as a complex process (Eynon, 2015). This can especially be a risk for neurodiverse children, who might function in diverse ways. Will wearables revolutionize our insight in learning processes, or will it lead to a universal template for learning with very little acceptance of deviation from the norm? Antle and Kitson (2021) point out that “most e-wearables, even those designed for children, were designed around normative assumptions and values often reflecting affluent, adult, male, and performance-oriented end-users” (p. 328). These norms can have a negative influence on young users, which is extra poignant because their identity is still developing (Antle and Kitson, 2021). Jovanovic and Kay (2023) warn that the use of wearable devices can result in altered and unhealthy perceptions of self-worth. In Drew and Gore's (2014) study on the use of wearables in an obesity prevention program for schools, they warn that students, when interpreting the data, might regard themselves abnormal, and feel pressured to conform to the group ideal (Drew and Gore, 2014). In discussing this study, Jovanovic and Kay (2023) emphasize that wearable data in the classroom should be used to promote a broader range of individualized learning experiences.

#### 4.2.2 Internalizing neo-liberal norms of productivity

Some worry that wearables might enforce a quantified self model, accompanied by internalizing neoliberal norms of productivity (Eynon, 2015; Lupton, 2016; Lupton and Williamson, 2017). In this tradition, individual wellbeing is often defined as higher performance and/or increased productivity, which is a biased assumption in that it focuses on one dimension of self-development only (Antle et al., 2022). Students might find it hard to switch of and relax, and live in the moment, as they might be constantly measuring themselves, and comparing themselves to others, resulting in stress and neglect of other activities (Parsons and Rosen, 2018; Antle and Kitson, 2021). Antle et al. (2022) found for example, in a critical design study, that 9–11 year old students thought that the quantified self movement influenced their agency: “If you took your limited steps that you do each day, but your watch says ‘no, you need to take more'. It's telling you what to do. It's making a choice for you.” (Antle et al., 2022). Baker (2020) warns that wearables might increase the feeling that we are never doing well enough.

#### 4.2.3 Quantified data as the primary source of self-hood?

Antle and Kitson (2021) worry that young people might increasingly view quantitative data as a primary source on their identity and self-hood, and that this might negatively influence the development of their personality. For example, one might feel less unique when one has average scores (Antle and Kitson, 2021). Many have outlined the effect that wearables and the quantified self-movement can have on peoples' self-concept. As Bower and Sturman (2015, p. 344) state: “More than just technical solutions, wearable technologies constitute a shift from computers as detached tools to technologies as embodied companions that become an extension of self.” Eynon (2015) worries that people might prioritize the quantified version of themselves instead of a more qualitative evaluation. The same can apply to teachers who might start over-relying on the quantified profiles of their students.

#### 4.2.4 Definition of a good teacher: technology before pedagogy?

There is also a risk that the parameters measurable through wearables increasingly determine who we perceive as good teachers and who not, hence losing sight on other important parameters. In their stakeholder research among 66 educators with affinity with wearable technology, Bower and Sturman (2015) found that some of their respondents worried that educators would put technology before pedagogy. Sandall (2016) also warns of the risk of implementing technology for the sake of technology. Implementing technology comes at a cost. Teachers have limited time and resources, using these technologies requires an investment of teachers, time they cannot invest in enhancing pedagogical skills and hence might negatively affect educational quality (Coffman and Klinger, 2015; Bower and Sturman, 2015). A worry related to this is that the implementation of these kind of technologies might cause a digital divide among teachers. In the selection of teachers, technology-wise teachers might be preferred over teachers who might be less familiar with technology, but have otherwise great pedagogical qualities (Borthwick et al., 2015; Sandall, 2016).

#### 4.2.5 Epistemic solutions to unjust self-redefining

An underlying concern of the issues mentioned above is the worry that quantitative data may become increasingly dominant in the evaluation and definition of what is normal or good, and that the normative process behind the analysis of the data, remains unnoticed. Data collected by wearables may be perceived as objective, however, setting parameters of normality is a normative process. One must guard who is in power of defining these, and ideally the interpretation of data happens in dialogue with those concerned. New technological data should be accompanied with normative discussions on what good parameters of education and pedagogy are. This concern regarding power and normalization is tightly connected to the concerns regarding the epistemic issues, explainability, and scientific rigor discussed earlier. Scientists and developers should be aware of the normativity behind categories, and should carefully monitor the effect their technology has on identity, and have normative discussions with the end users about the right parameters. When end-users understand the explainability of the technology, they are less tempted to jump to hasty conclusions.

### 4.3 Liberating responses, evading power

Foucault has become most known for his analysis of oppression, governance, surveillance and punishment, and how people internalize external and possibly oppressive norms in how they define themselves. However, at the end of his life, Foucault became intrigued by the often unexpected ways in which people can respond to governance and overturn power relationships. He calls this “technologies of the self,” but basically this involves all the responses people exhibit to reclaim their identity, by defining themselves through inside norms rather than outside ones, and to evade power or overturn power relationships (Foucault, 1988). These are ways in which we recover our private space, and defend those parts of us that do not fit within the normalization processes (Hernández-Ramírez, 2017; Agamben, 2009). This interesting dimension of Foucault's philosophy shows that power is not a one-way, top down relationship, but fluid. Power happens in relationships rather than being a property of one group that governs another group.

For example, although there is still great technological illiteracy among young and old, in general young generations feel more at ease with new technologies. Students might outsmart the teachers and use the technology in a way unintended by the teachers or schools. It is implicitly assumed that those in power control these technologies, however, the current generation of students is more at ease with these technologies than the average teacher (so called digital natives vs. digital immigrant) (Prensky, 2001).

Unfortunately, the literature focuses most on oppression and power and the influence on identity, and less on the liberating responses and the overturning of power relationships. In the discussion of Section 5, we elaborate more on how end-users can be involved in the development and implementation of technology, so that their innovative perspectives can be incorporated.

## 5 Discussion and conclusion: lessons learned for an ethical implementation of wearables in the classroom

In this study we examined ethical considerations regarding wearables for tracking mental states, like attention and stress, in the classroom. We distinguished three angles through which ethical analysis can be conducted: epistemic, principle-based, and Foucauldian. The epistemic ethical approach (e.g., Maxwell and Racine, 2011) focuses on the implications of the scientific rigor, such as reliability (consistency) and accuracy (precision), of any emerging technology and argues that epistemic and ethical issues are closely intertwined. Most importantly, this approach aims to assess whether end-users have a meaningful understanding of the technology they are using, including an adequate understanding of the technology's limitations regarding, e.g., informativity and explainability (Mecacci and Haselager, 2017). The principle-based ethics angle (e.g., Beauchamp and Childress, 2002) assesses new technologies against universal ethical principles like beneficence (doing good), non-maleficence (to do no harm), autonomy, justice, (mental) privacy, and consent. A Foucauldian ethics angle argues that new technologies influence power relationships and identity formation. Technologies can be oppressive, but people also have unexpected answers to technologies in which they overturn those power relationships.

A focus group of high school students was set up to identify young people's views on such wearable technologies and allow for an initial reflection on the theory-based ethical considerations. The input from the focus group, showed the merits of a Foucauldian angle, so for the remainder of the paper we focused on a Foucauldian ethical analysis.

Our Foucauldian analysis shows that although wearables may provide important information on learning and attention, and although users are enthusiastic about the potential, there are several risks regarding governance and surveillance, normalization and exclusion, placing technology before pedagogy, stimulating neoliberal values and quantified self-understanding, and possible negative impact on identity for those who think they are outside of the norm.

The focus group's remarks underscored some of the worries about mental privacy, self-image and stress. However, in general the students were curious and positive about the technology. The students nicely illustrated under which conditions, and for which groups the wearables could provide useful information. For inexperienced teachers, the technology could support them to improve their pedagogical qualities. Experienced teachers would not need this technology. Malevolent teachers may use wearables to humiliate students.

The high school students demonstrated that people are not only subjected to new technologies, but also adjust them to their own goals. According to the students, data collected by wearables does not so much give insight into students' capabilities, but rather regarding teachers' flaws. In addition to being concerned about being potentially governed by wearables, they also suggest ways to invert the power relationships and apply the technology to govern teachers. Mostly, the students demonstrated that end-users have sensible and unexpected ideas about the added value and ethical risks of technologies within a specific context. The data shows that students did not feel too vulnerable with regard to new technologies, but rather have ideas on how the technology can be used to their benefit.

Below, we suggest different lessons that can be learned from our investigations for an ethical implementation of wearable technologies in the classroom.

### 5.1 Prohibition? The EU Artificial Intelligence Act

Our analysis of the risks of governance, surveillance and punishment, and a negative effect on self-image, might suggest that we should reject such wearable technology in the classroom altogether. Radical Foucauldians would indeed suggest that. Overall rejection of the technology may also be related the EU Artificial Intelligence Act that entered into force on 1 August 2024 and that could be taken to prohibit emotion recognition systems in education. Alternatively interpreted, the act places emotion recognition systems in the “high risk” category given “the limited reliability, the lack of specificity and the limited generalisability” which “therefore” “may lead to discriminatory outcomes and can be intrusive to the rights and freedoms of the concerned persons” (recital 44). Concerning the prohibition regarding these systems in education, recital 44 of the act further states that “Considering the imbalance of power in the context of [work or] education, combined with the intrusive nature of these systems, such systems could lead to detrimental or unfavorable treatment of certain natural persons or whole groups thereof.” While attention is usually not considered an emotion, and the act did not include it in a statement that comes closest to a definition (recital 18: “The notion refers to emotions or intentions such as happiness, sadness, anger, surprise, disgust, embarrassment, excitement, shame, contempt, satisfaction, and amusement”), attention monitoring in classrooms is close in the least.

The Act certainly raises important points, however, our analysis shows that these risks could also be mitigated. With regard to reliability and generalizability, we showed that an awareness of epistemic issues and specifically the importance of explainability could limit negative risks. With regard to the power balance in the classroom the students in our focus group showed that the power-relations are not as straight forward as is often assumed. Also, some of the issues raised could be mitigated by technology. European Privacy legislation today has instruments allowing a management of measurements from wearable technology that guarantees anonymity; and ensure privacy. In addition, computer science can generate systems that protect privacy and are aimed at improving the management of attention and stress rather than aimed at judgment and control. Also, the development of empirical evidence-based guidelines on ethical issues could mitigate risks while ensuring the positive sides (Tzimas and Demetriadis, 2021).

Let us return to Jinhua Xiaoshun county primary school (Tai, 2019), which used all kinds of wearables in the classroom. After the report in the Wall Street Journal, the school faced so much backlash, that they decided to halt the trial. However, the school stated that the report exaggerated the situation. Data was not shared with parents, and the technology was nowhere near the mind reading properties that the media assumed it had. The technology simply helped to gain more insight into attention of students. They also added that the preliminary analysis showed that its usage (30 min, twice a week) had a positive effect on attention, and that students, parents and teachers were positive. Yet, due to the media pressure, they decided to halt the trial. Although we do not have any first hand data to make a good assessment of the ethical aspects of this case, it demonstrates the public fear for these types of technology.

Our Foucauldian analysis show that new technologies often have unexpected effects on how we perceive ourselves and the world. It would be most safe to prohibit technologies that can have any such effects. However, the downside of prohibiting new technologies before they are even implemented, is that their benefits will never be known. Wearables have the potential to give important insights into learning and teaching, and might improve personalized learning. With emerging technologies, not all risks and affordances can be anticipated, and sometimes practicing ethics requires taking a vigilant leap forward. The balancing act between protection and curiosity might be hard, but has advantages over prohibition. Below we describe some recommendations for an ethical implementation of wearables in the classroom, to explore the added value of such devices, and their experienced down-sides in real life.

### 5.2 Co-creation between scientists, end-users and ethicists

The ethical analysis of power, relationships and identity, showed that people are not as much victims of technologies, as some suggest, but can also negotiate and overturn their use. Students can be seen as vulnerable and in need of protection, but they can also be seen as having an important voice, and important insights about how, when and for whom wearables could be implemented. End-users should be involved in the implementation process of wearables. Such an approach would treat them as autonomous subjects, rather than vulnerable people.

Young people might hold different views than the adults making regulations. Engen et al. (2017) describe how the students, when they received the wearables during the research, were excited and highly motivated to use them. This indicates the popularity of wearable technology among students. Students are often more technology oriented than the previous generation. Both Demir and Demir (2017) as Ensmann (2020) show that students already have integrated a number of wearable technologies into their daily routine, and that they also bring these technologies to the classroom. Examples are fitbits and smartphones that measure physical activity, sleep cycles, heart rate, and etcetera.^3^ However, Engen et al. (2017) also outlined that students seemed largely unaware of the ethical aspects of the wearables they already used. Hence, caution is needed. Ethicist could coach this process of co-creation between scientists, students, teachers, and parents, in the implementation of wearables.

### 5.3 Training in ethical risks, the normativity of science, and explainability

Some suggest that rather than prohibiting new technologies, it is crucial to train people regarding their risks. Awareness of risks of classroom digitalization should be part of the education curricula for students, parents and teachers alike (Engen et al., 2017; Borthwick et al., 2015; Agesilaou and Kyza, 2022; Antle et al., 2022). Antle et al. (2022) suggest that we should support young people's computational empowerment: “when students can recognize the ethical choices embedded in technology and reflect on the consequences of these choices for the people who will use the technology, including themselves.” They developed a wearable design workshop for young people (9–11 years old), which also used ethics cards to challenge them to think of the ethical aspects of the technology. Agesilaou and Kyza (2022) also urge to develop more educational material to support students in critical thinking skills to “how children can be empowered through scaffolded inquiry experiences to ‘reflect on their own use of smart, self-tracking devices and gain a deeper understanding of the digital infrastructure and the political economy of digital data.' These skills can help students to resist technology driven oppression and develop a healthy self-image. Learning to work with technologies is also expected to teach users about the technologies” limits, and explainability, so users can use it in an effective and responsible way. In line with this, scientists should be aware of the normativity that can lay behind apparent objective categories of normalization.

### 5.4 The importance of normative discussions on good pedagogy

The issues raised in our focus group underscore the importance of a normative discussion between different parties at a high school (teachers, students, and parents) and those who develop wearable technologies to track mental states, during the process of development and before implementing new technologies. These discussions should also involve normative analysis of what good teaching is, which characteristics determine what a good teacher is, and what a good pedagogical climate is for students. New technologies can provide interesting data, but how this data is interpreted is of the highest importance, and the evaluation of good teaching should not be reduced to one parameter.

We would like to conclude with the words of Dorrestijn (2012): “Technology is not set in opposition to human freedom and morality; rather coping with the influences of technology is seen as part of becoming a moral subject. The ethics of technology developed after Foucault focuses on care for the quality of our interactions and fusions with technology. (…) it is not to be rejected, neither is it the greatest danger, but it does deserve the greatest care.”

## Data Availability

The raw data supporting the conclusions of this article will be made available by the authors, on reasonable request.

## Footnotes

^1^We have adjusted Foucault's heavily philosophical jargon to concepts that are more common for non-philosophy readers. Foucault uses the language of: governance/governmentality, subjectification, and technologies of the self.

^2^The article presents the situation as a specifically Chinese problem, because of the lack of (mental) privacy and data protection regulation, however, a representative of the school states that the wearables were a gift of a former student who studied in the US, and saw a widely use of wearables there (https://www.theguardian.com/world/2019/nov/01/chinese-primary-school-halts-trial-of-device-that-monitors-pupils-brainwaves). There might be a cultural bias where Western media are more critical of the use of wearables in China than in Western countries.

^3^There might arise a tension between wearables that students themselves bring to the class, and the wearables the teachers implement (Sandall, 2016).
