# Supplementary material for: Wearables for tracking mental state in the classroom: ethical considerations from the literature and high school students
Source: Front Neuroergon. 2025 May 13;6:1536781. doi: 10.3389/fnrgo.2025.1536781 (PMC12106337; doi:10.3389/fnrgo.2025.1536781)
Supplement: Supplementary file 1 [file Data_Sheet_1.docx]

## Appendix: Overview of ethical issues identified in the literature

| **Ethical risk** | **Reference** |
| --- | --- |
| **1. PRINCIPLES** |  |
| **Equality and justice** |  |
| - Costs of wearables, affordability | Attallah 2018; Bower 2015; Motti 2019 |
| - Access by students of all socioeconomic levels (inequalities; economic divide) | Borthwick 2015; Motti 2019 |
| - Are schools obliged to invest in this? | Sandall 2016 |
| **(Mental)Privacy and security** |  |
| - Privacy and security | Attallah 2018; Bower 2015; Borthwick 2015; Motti 2019; Demir 2016 |
| - Mental privacy | Mecacci and Haselager 2019 |
| - Data storage and access: Dependence on outside vendors for storage, access and analysis of big data. | Borthwick 2015; Motti 2019 |
| - Teachers are not trained in these risks. | Borthwick 2015 |
| - Student safety: Abuse of data by others | Demir 2016; Janssen 2021 |
| - Student safety : Abuse of data by students themselves. Students are not aware of privacy risk. They might post sensitive information about themselves. | Borthwick 2015  Engen 2017 |
| - Intellectual property; data ownership | Demir 2016; Motti 2019 |
| - Legal issues | Bower 2015; Borthwick 2015 |
| - Overselling by commercial parties | Janssen 2021 |
| **Consent** |  |
| - What if, unintendedly wearables can function as a diagnostic instrument? There is no consent for that. | Borthwick 2015 |
| **Autonomy** |  |
| - Wearables can dictate norms to students that they would otherwise not adhere to. | Antle et al. 2022 |
|  |  |
| **2. EPISTEMIC** |  |
| **Scientific rigourness** |  |
| - Low reliability (consistency), accuracy (precision) and informativity. | Mecacci and Haselager 2019 |
| - Low accuracy | Demir 2016 |
| - Overselling or underselling | Wexler 2019; Wexler and Thibault 2019 |
| - Lack of communication between scientists and end-users | Brouwer 2021 |
| - Failures in explainability | Khosravi 2022 |
| - large data sets, which can be overwhelming for posterior analysis. | Motti 2019 |
| **Technical issues** |  |
| - Implementation issues | Demir 2016 |
| - Accessibility. Technical problems and infrastructure (network connectivity and battery life). Issues with connectivity and compatibility. | Attallah 2018; Bower 2015; Motti 2019; Demir 2016 |
| - Lack of technical support in case of problems | Bower 2015 |
| - Development of software. Hardware as well as software malfunctions can impair the learning experiences of students | Bower 2015; Motti 2019 |
| - Classroom limitations | Borthwick 2015 |
| - problems to learn how to efficiently use the device and application (need for high usability levels in order to ensure acceptance and sustain adoption) | Motti 2019 |
| - The volatility of the wearable market which can challenge sustained adoption of wearable solutions | Motti 2019 |
|  |  |
| **3. FOUCAULDIAN** |  |
| **Technology before pedagogy** | Bower 2015 |
| - Overreliance on technology which is obstructing independent thinking | Attallah 2018 |
| - Distraction by the devices which negatively impact the quality of learning. | Attallah 2018; Bower 2015; Motti 2019 |
| - Overreliance on technology to support cognition could lead to a deterioration of some skills (social intelligence) | Bower 2015 |
| - Cognitive overload for teachers and students due to multitasking | Bower 2015; Motti 2019 |
| - The need to familiarise oneself with the interface may impact on the educational quality | Bower 2015; Coffman and Klinger 2015 |
| - Wearables may not have sufficient pedagogical value | Attallah 2018 |
| - Implementing technology for the sake of implementing technology. | Sandall 2016 |
| - Dependence on technology | Motti 2019 |
| **Digital divide** |  |
| - Digital divide/gap: Demands computing skills of both lecturer and students. | Borthwick 2015; Sandall 2016; Demir 2016; Attallah 2018 |
| - Teachers and students who resist change might be disadvantaged | Attallah 2018 |
| **Quantified self** |  |
| - Negative self-understanding, especially problematic for the developing sense of identity | Antle et al 2022; Jovanovic and Kay 2001; Drew and Gore 2016 |
| - Anxiety among students. Students fear the constant recording of their actions; therefore brief trainings are recommended to reduce students’ anxiety. | Motti 2019 |
| - Authenticity may be challenged by biowearables that interrupt enjoyment that comes from being present in the moment | Antle et al 2022 |
| - Conflict with wearables students themselves bring into the classroom. | Sandall 2016 |

Table 2: overview of ethical issues identified in the literature

## Appendix: presentation of wearables for the students


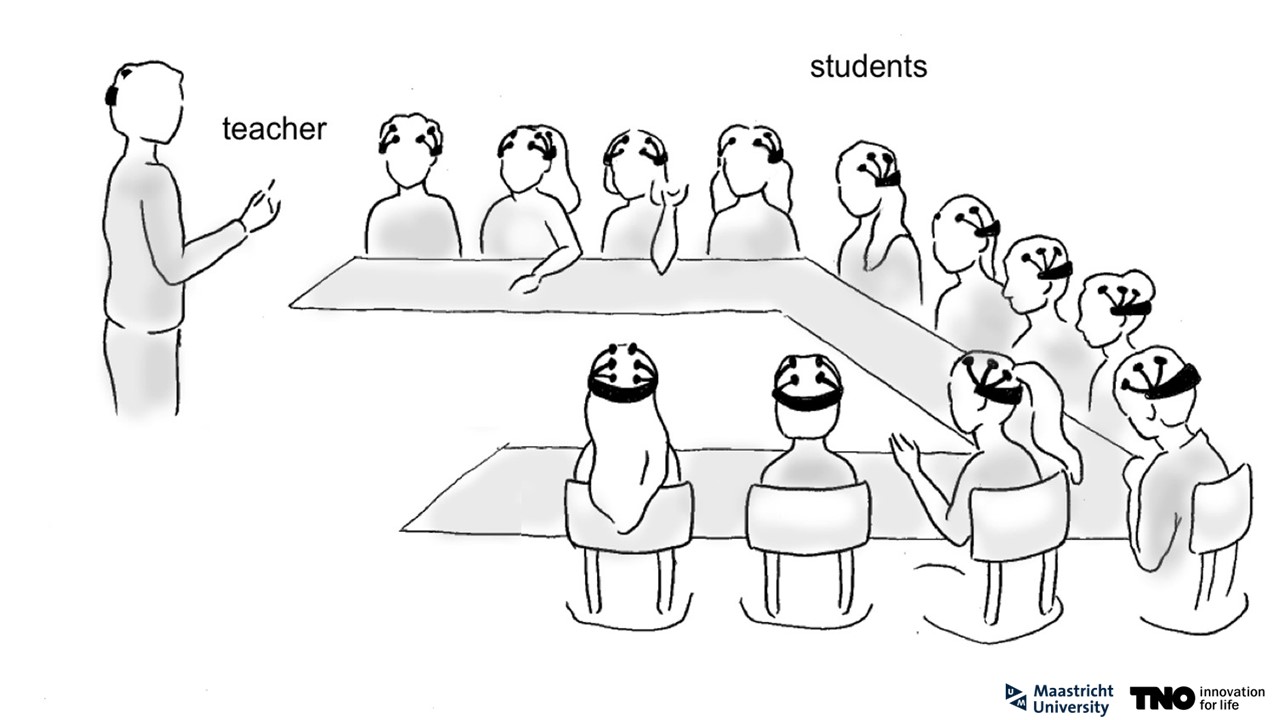


Copyright: Ivo V. Stuldreher


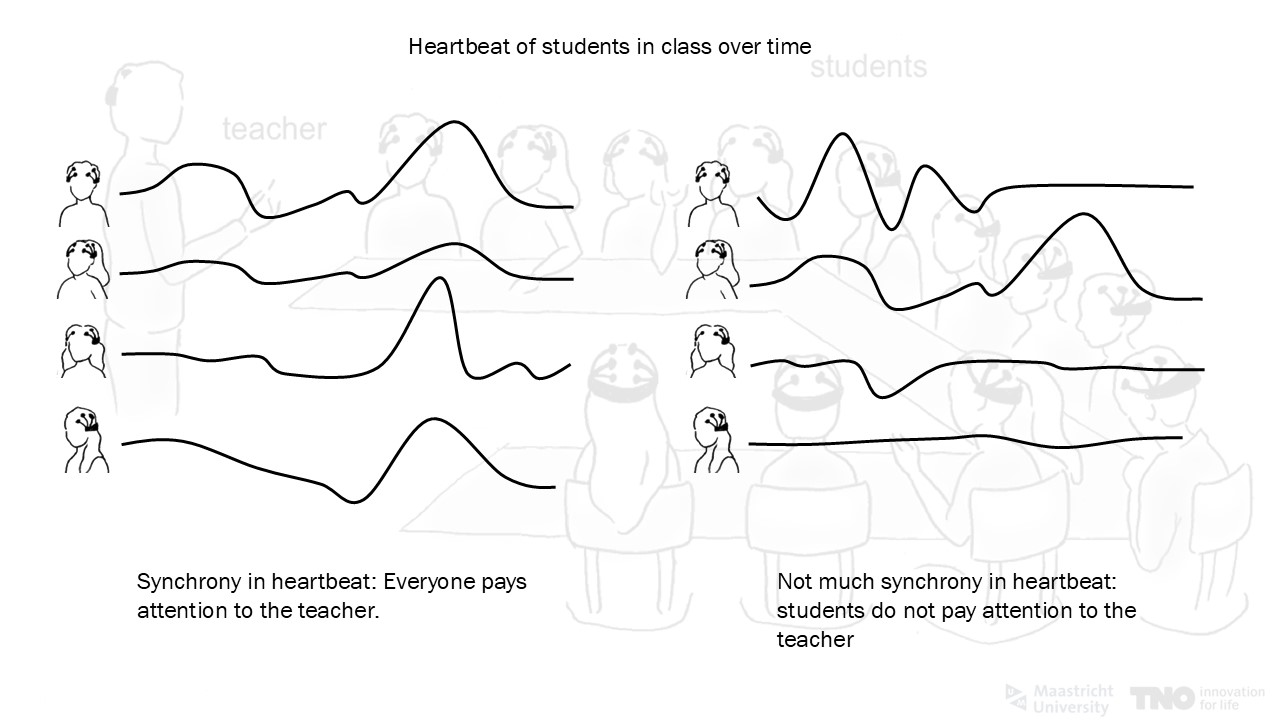


Copyright: Ivo V. Stuldreher
